# The LuxS/AI-2 Quorum-Sensing System Regulates the Algicidal Activity of *Shewanella xiamenensis* Lzh-2

**DOI:** 10.3389/fmicb.2021.814929

**Published:** 2022-01-28

**Authors:** Jian Liu, Kaiquan Liu, Zhe Zhao, Zheng Wang, Fengchao Wang, Yuxiu Xin, Jie Qu, Feng Song, Zhenghua Li

**Affiliations:** ^1^Shandong Key Laboratory of Biophysics, Institute of Biophysics, Dezhou University, Dezhou, China; ^2^School of Bioengineering, Qilu University of Technology, Shandong Academy of Sciences, Jinan, China

**Keywords:** quorum-sensing, algicidal bacteria, LuxS/AI-2, *Shewanella xiamenensis*, bloom control

## Abstract

Cyanobacterial blooming is an increasing environmental issue all over the world. Algicidal bacteria are potential tools for the control of algal blooms. The algicidal activity in many bacteria exhibits quorum-sensing (QS) dynamics and the regulatory mechanism of this activity in these bacteria is unclear. In this study, combining genomic sequencing and genome editing, we have identified that the primary quorum-sensing system in the isolated algicidal strain *Shewanella xiamenensis* Lzh-2 is the LuxS/AI-2 signaling pathway. Disruption of the QS system through recombination deletion of the LuxS gene led to a loss of algicides production and algicidal activity. Restoration of the LuxS gene in the deletion mutant compensated the QS system and recovered the algicidal activity. Consequently, we proved that Lzh-2 regulates the algicidal activity through LuxS/AI-2 quorum-sensing system.

## Introduction

Cyanobacterial blooms, resulting from eutrophication, have substantial harmful effects such as animal deaths and human illness (Huisman et al., 2018). There is a huge need for eco-friendly algal bloom control methods. Algicidal bacteria that decrease the growth of cyanobacteria by producing algicides is a potential option (Yang et al., 2020). To date, many species with algicidal activity have been reported, such as *Rhizobium* strain AQ_MP (Pal et al., 2021), *Shigella* sp. H3, *Alcaligenes* sp. H5 (Xue et al., 2021), *Paracoccus homiensis* (Ding et al., 2021), *Enterobacter* sp. EA-1 (Lu et al., 2021), etc.

Lzh-2 was an efficient algicidal bacterium isolated from Taihu Lake (Li et al., 2014), where *Microcystis aeruginosa* is the dominant bloom-forming cyanobacterium (Ye et al., 2011). Our previous study showed that the main algicidal substances of Lzh-2 were hexahydropyrrolo[1,2-a]pyrazine-1,4-dione (S-2A) and 2, 3-indolinedione (isatin, S-2B) (Li et al., 2014). Interestingly, Lzh-2 exhibited a cell density-dependent behavior that algicidal compounds were produced only when the density exceeded a threshold of about 10^8^ cells⋅mL^–1^. This behavior was called quorum-sensing (QS). Some algicidal bacteria exhibited the QS behavior (Dow, 2021).

Quorum-sensing relies on the communication between cells through signaling molecules. There are two well-characterized QS signaling systems: N-acylhomoserine lactones (AHLs) and Autoinducer 2 (AI-2) signaling (Dow, 2021). LuxI and LuxR are the crucial genes in the AHLs system. LuxI participates in the synthesis of AHLs, while LuxR mediates the transmission of signals from external AHLs to related biological activities (Cui and Harling, 2005). The AHLs and LuxI/LuxR QS system has been identified in several algicidal bacteria (Guo et al., 2016; Zhang et al., 2021). In contrast, LuxS, the core gene of the QS system, participates in the synthesis of AI-2, and the signals are transmitted through different regulators (Zhao et al., 2018). Few LuxS/AI-2 QS system cases in algicidal bacteria have been reported (Zhang S. J. et al., 2020).

In the present study, we used genome sequencing and genome editing strategies to identify the primary QS system in the Lzh-2 strain and reveal how it regulates the algicidal activity through quorum sensing.

## Materials and Methods

### Strains, Plasmids, and Culture Conditions

All strains and plasmids used in this study are listed in Supplementary Table 1. All primers were synthesized by Sangon Biotech (Shanghai, China) and listed in Supplementary Table 2. All bacteria strains were cultivated aerobically in Luria-Bertani (LB) medium (Difco, Detroit, MI) at 30°C (*Shewanella xiamenensis*) and 37°C (*Escherichia Coli*), respectively. Where needed, the growth medium was supplemented with chemicals at the following concentrations: 2,6-diaminopimelic acid (DAP), 0.3 mM; ampicillin (Amp), 100 μg/mL; kanamycin (Kan), 50 μg/mL; and gentamicin (Gm), 15 μg/mL. *Microcystis aeruginosa* 9,110 was grown in BG11 medium at 25°C under 40 μmoL photons/(m^2^⋅s) and a 12 h:12 h (light:dark) cycle (Tian et al., 2012). Cell densities were quantified using a hemocytometer under a light microscope (magnification × 400). All chemicals were purchased from Sigma-Aldrich (Shanghai, China) unless otherwise noted. All experiments were repeated at least 3 times.

### Genome Sequencing and Analysis

Total DNA was extracted using the E.Z.N.A.^®^ Bacterial DNA Kit (Omega Bio-Tek) according to the manufacturer’s instructions. The quality of the extracted DNA was verified through agarose electrophoresis and fluorometrically qualified with the Qubit dsDNA HS kit (Thermo Fisher Scientific) through Qubit Flex Fluorometer (Thermo Fisher Scientific). Two libraries were constructed separately. Paired-end (PE) library with an inserts size of 300 bp was constructed with TruSeq™ DNA Sample Prep Kit (Illumina). The library was sequenced using Illumina NovaSeq 6,000 (Illumina) with 150 PE reads at Shanghai Personal Biotechnology Co., Ltd. (Shanghai, China). The Pacbio library with an inserts size of 20 kb was constructed using the Template Prep Kit 1.0 (Pacbio) and sequenced with the PacBio Sequel (Pacbio) apparatus at the above place. The obtained sequence was uploaded to the NCBI Sequence Read Archive repository (accession number PRJNA779223).

All reads were quality-filtered. The results from Pacbio Sequel were assembled by HGAP (Chin et al., 2016). Then the complete genome was obtained by error correction with reads from PE library using Pilon (Walker et al., 2014). The complete genome sequences have been submitted to the genebank database of NCBI with the accession number NZ_CP069350.1. GeneMarkS (Besemer et al., 2001), Barrnap, and tRNAscan-SE (Lowe and Eddy, 1997) were used to predict open reading frames and non-coding RNAs. The function of the predicted genes was obtained by aligning the ORFs with the NR database. The circular genome visualization was generated with CGView (v2.0). The taxonomy classification of Lzh-2 was determined by GTDB-tk (Chaumeil et al., 2020). LuxS DNA sequences of other organisms were obtained from GenBank (Supplementary Table 3). The phylogenetic tree was constructed by MEGA11 (Tamura et al., 2021) using the Neighbor-joining algorithm with default parameters.

### AI-2 Bioassay

The AI-2 bioassay was modified from Bodor et al. (2008). *Vibrio harveyi* BB152 ATCC^®^ BAA1119 and *V. harveyi* BB170 ATCC^®^ BAA-1117 (Bassler et al., 1993) were used to detect AI-2. *V. harveyi* BB170 was a biosensor for AI-2 only, while *V. harveyi* BB152 was used as a positive control. 1 mL culture supernatant of *Shewanella xiamenensis* strains was sampled and concentrated by centrifugation (13,000 rpm, 2 min) and filtration (0.22 μm) into 100 μL stock. The sensor strain *V. harveyi* BB170 was freshly inoculated and incubated (160 rpm, 30°C) to an OD_600 *nm*_ of 1.0. 180 μL of 5,000 times diluted *V. harveyi* BB170 culture was mixed with 20 μL samples in a microplate. The plates were incubated at 30°C with shaking at 600 rpm for 4 h. The luminescence of the samples was recorded by a multi-channel microplate reader (HBS-1096, DeTie). The percentage of AI-2 activity was calculated as the ratio of the test sample divided by the positive control (*V. harveyi* BB152).

### In-Frame Deletion and Complementation

We applied the Fusion PCR method (Gao et al., 2006) to construct the in-frame deletion strains of LuxS. In brief, two fragments flanking LuxS were amplified independently first and joined together by overlap PCR. The resulting fusion fragment was introduced into the plasmid pDS3.0 through the In-Fusion HD cloning kit from Takara Bio (Kusatsu, Japan). The resulting plasmid was transformed into *E. coli* WM3064 (Saltikov and Newman, 2003), and then transferred into *S. xiamenensis* by conjugation. Integration of the plasmids on the chromosome was selected by gentamycin resistance and confirmed by PCR. The transconjugants were grown in LB broth without NaCl and plated on LB plates containing 10% sucrose. Sucrose led to the deletion of the integrated plasmid. Colonies without the plasmid sequence were selected by gentamycin-sensitive and sucrose-resistant phenotype. Half of these colonies contained the complete LuxS gene, while the other half contained the deletion mutants. They were verified through PCR and partial sequencing. The generation of the deletion mutant was illustrated in Supplementary Figure 1.

For the complementation of LuxS in the deletion mutant, LuxS and its native promoter were amplified by PCR and integrated with pBBR1MCS (Kovach et al., 1995) through the In-Fusion HD cloning kit (Takara Bio). The achieved plasmid was transformed to the deletion mutant by mating with *E. coli* WM3064 containing the vector. The presence of the plasmid with LuxS was further confirmed by plasmid extraction and partial sequencing. The generation of the complementation stain was illustrated in Supplementary Figure 2.

### Quantification of S-2A and S-2B by LC-MS

Culture supernatants of *S. xiamenensis* were sampled and mixed with an equal volume of ethyl acetate. After keeping the mixture in a separation funnel for 24 h, the ethyl acetate layer was collected and evaporated. The resulting materials were dissolved in 1 mL of water and filtered through a 0.22 μm membrane filter. The filtrates and standard solutions of S-2A and S-2B were subjected to LC-MS analysis in positive mode. MS data were acquired and processed using the LC-MS Qualitative Analysis B.04.00 software supplied with the instrument. Using this program, mass chromatograms corresponding to S-2A and S-2B were extracted and integrated from the total ion chromatogram. The concentrations of S-2A and S-2B in the filtrate were determined by comparing the peak areas of S-2A (or S-2B) with those of the standards (Armando et al., 2012).

### Statistical Analysis

Data are presented as mean ± standard deviation of triplicate cultures. Statistical analyses and figures were performed with Origin 8.5 (Origin Lab Corporation, United States) software. A two-way *t*-test was used to analyze the significance level, and a *p*-value < 0.05 was considered statistically significant.

## Results

### Genome Sequencing of the Strain Lzh-2

**FIGURE 1:**
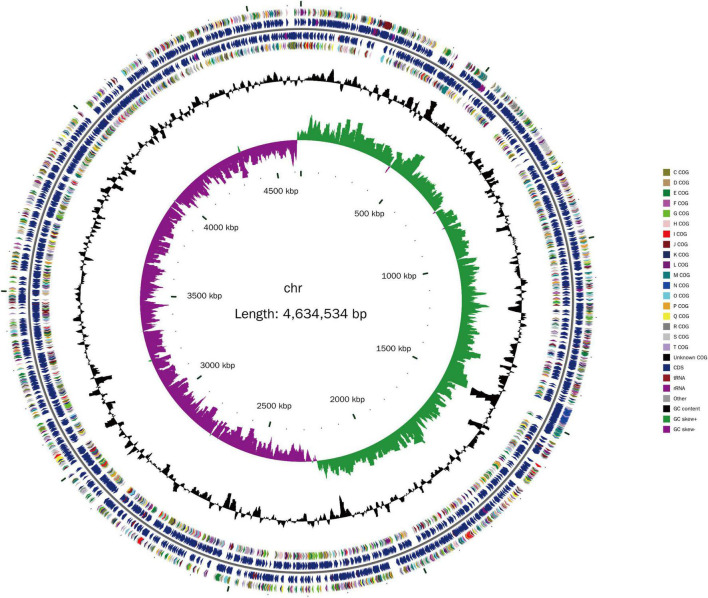
The genome structure of Lzh-2. The inner circle is the scale label. The second circle represents the GC skew (green positive, purple negative). The third circle represents the GC content. The 4th and 7th circles represent the COG classification. The 5th and 6th circles represent the genes identified. Different colors denoted the COG classification.

The isolated Lzh-2 stain was sequenced by a combined strategy of next-generation sequencing and PacBio long reads sequencing. The complete genome was obtained with a total length of 4.6 Mbp (Figure 1) and an average GC content of 46.31%. A total of 4,023 protein-coding genes and 310 RNA-coding genes were predicted in the genome. By comparing the genome of Lzh-2 with known genomes, it was classified as *Shewanella xiamenensis*.

### Identification of the LuxS/AI-2 System in Lzh-2

Our previous study showed that Lzh-2 exhibited quorum-sensing behavior (Li et al., 2014). To explore the mechanism, we firstly screened the genome of Lzh-2 for known quorum-sensing genes. We found the existence of the LuxS gene in Lzh-2, which is the crucial gene for the AI-2 quorum-sensing system. LuxS from Lzh-2 and *S. xiamenensis* showed 99% identity with only three different nucleotides, which were synonymous mutations (Supplementary Figure 3).

**FIGURE 2:**
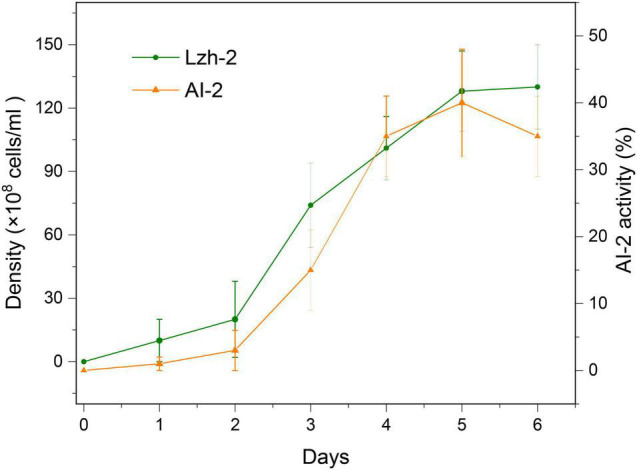
The density of the Lzh-2 strain (green) and the activity of AI-2 produced (orange).

To further confirm the activity of this quorum-sensing system, we tested the density dynamics of Lzh-2 and the related activity of AI-2 in Lzh-2 (Figure 2). The results showed that AI-2 activity was positively correlated with the density of Lzh-2 and then deceased when Lzh-2 reached the stationary phase.

### Disruption of LuxS Suppresses the Quorum-Sensing Behavior and Algicidal Activity

**FIGURE 3:**
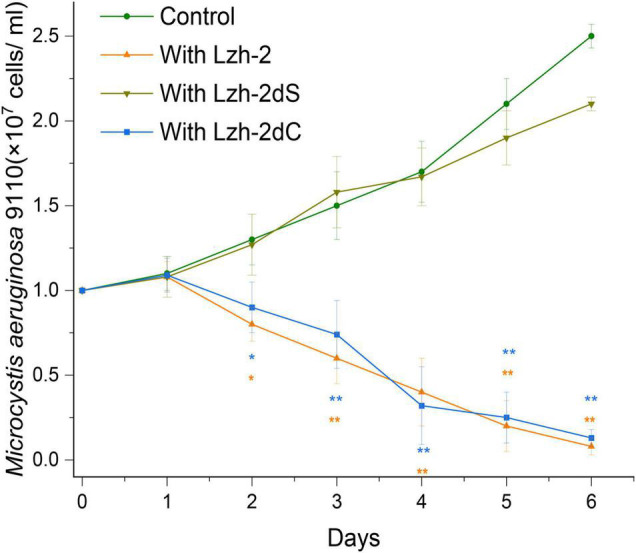
Dynamics of cell density of *Microcystis aeruginosa* 9110 co-cultured with or without *Shewanella xiamenensis* strains (1:1). Control without co-cultures (Green); co-cultured with strain Lzh-2 (orange); co-cultured with strain Lzh-2dS (dark yellow); co-cultured with strain Lzh-2dC (blue). * and ** represent *p* < 0.05 and *p* < 0.01 comparing to the control group, respectively.

Lzh-2 had significant algicidal activity, as shown in Figure 3. The density of *Microcystis aeruginosa* 9110 decreased when co-cultured with Lzh-2. To study the relationship between quorum-sensing and algicidal activity, we completely deleted the LuxS gene from the Lzh-2 genome by two-step recombination, resulting in the strain Lzh-2dS. LuxS deleted stain didn’t show the algicidal activity, and *Microcystis aeruginosa* 9110 grew normally with Lzh-2dS (Figure 3). To further confirm the function of LuxS, the whole LuxS cassette was reinserted into Lzh-2dS, which was named Lzh-2dC. Lzh-2dC restored the algicidal activity that *Microcystis aeruginosa* 9110 could not grow with Lzh-2dC (Figure 3).

**FIGURE 4:**
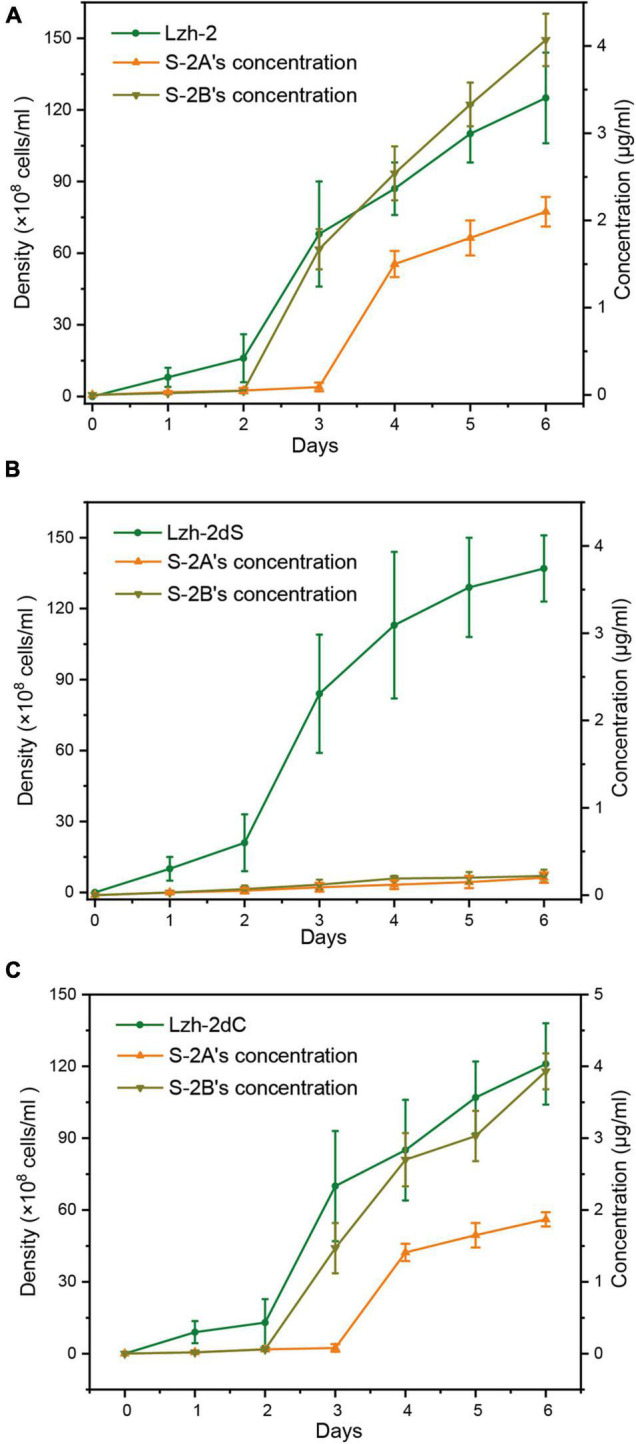
Density of different strains of *Shewanella xiamenensis* (green) and the corresponding concentration of S-2A (orange) and S-2B (dark yellow). **(A)** Lzh-2 strain; **(B)** Lzh-2dS strain; **(C)** Lzh-2dC strain.

The algicidal products of Lzh-2 were hexahydropyrrolo[1,2-a]pyrazine-1,4-dione (S-2A) and 2, 3-indolinedione (isatin, S-2B). The production of S2-A and S2-B was positively correlated with the density of Lzh-2 (Figure 4A), while the deletion of LuxS led to deficient production of S2-A and S2-B even at a high cell density of Lzh-2dS (Figure 4B). However, the complementary expression of LuxS in Lzh-2dC restored the production of S2-A and S2-B at high cell density (Figure 4C).

## Discussion

*Shewanella xiamenensis* is a widely spread species in many environments such as water, soil, and animal guts (Nguyen et al., 2020). The genome size and the average GC content of Lzh-2 are similar to other reported strains of *S. xiamenensis* (Leangapichart et al., 2021). *S. xiamenensis* is an important antibiotic resistance gene (ARG) harboring species that contains genomic *bla*_*OXA*_ genes, which encode a lactamase conferring resistance to carbapenem (Nguyen et al., 2020). The isolates from the hospital and human gut contain plasmids carrying transposon and ARGs, which may transmit ARGs to other bacteria (Yousfi et al., 2017; Leangapichart et al., 2021). In contrast, Lzh-2 has the genomic *bla*_*OXA*_ but not the plasmids. Thus, the risk of Lzh-2 transmitting ARGs is low.

Quorum-sensing has been observed in the genus *Shewanella* and plays a crucial role during biofilm formation and interaction with other species (Zhu et al., 2015; Mukherjee et al., 2020). Two quorum-sensing systems were identified in *Shewanella* sp. The quorum-sensing molecules AHLs have been identified in certain *S. xiamenensis* strains (Liébana et al., 2016), but most of these strains didn’t have the essential genes LuxI or LuxR (Sugita et al., 2017). Lzh-2 didn’t contain the LuxI gene, which participates in the synthesis of AHLs. However, LuxS, a crucial gene for the conversion of AI-2 signal molecules, was presented in Lzh-2. Our results showed a close positive correlation between cell density and AI-2 activity in the lag and exponential stage and a decrease of AI-2 activity during the stationary stage. This dynamic was observed in other *Shewanella* species which exhibit a LuxS/AI-2 quorum-sensing system. For example, the AI-2 activity in *Shewanella baltica* improved with the increase of the cell density, which was the critical component of QS system during biofilm formation (Zhu et al., 2015, 2016). Collectively, the primary quorum-sensing system in Lzh-2 is the LuxS/AI-2 signaling pathway.

The algicidal activity of Lzh-2 is closely related to quorum-sensing. The disruption of LuxS led to a loss of algicidal activity, while the restoration of LuxS compensated for this function. The relationship between quorum-sensing and algicidal activity has been observed in many different species (Chi et al., 2017; Wu et al., 2017). However, most of them are LuxI/LuxR quorum-sensing systems involving AHLs molecules (Guo et al., 2016; Zhang C. et al., 2020). Our previous study proved that the algicidal chemicals in Lzh-2 were S2-A and S2-B (Li et al., 2014). We showed that the disruption of LuxS can also decrease the activity of S2-A and S2-B, which could be compensated by the restoration of LuxS. Consequently, we proved that the algicidal activity of Lzh-2 is regulated by the LuxS/AI-2 quorum-sensing system by inducing the production of algicides S2-A and S2-B.

## Data Availability Statement

The data presented in the study are deposited in the NCBI repository, accession number PRJNA779223 (sequencing) and NZ_CP069350.1 (genome).

## Author Contributions

JL and ZL designed the research. KL, ZZ, ZW, FW, YX, and JQ performed the experiments. JL, KL, FS, and ZL analyzed the data. JL, FS, and ZL wrote and revised the manuscript. All authors contributed to the article and approved the submitted version.

## Conflict of Interest

The authors declare that the research was conducted in the absence of any commercial or financial relationships that could be construed as a potential conflict of interest.

## Publisher’s Note

All claims expressed in this article are solely those of the authors and do not necessarily represent those of their affiliated organizations, or those of the publisher, the editors and the reviewers. Any product that may be evaluated in this article, or claim that may be made by its manufacturer, is not guaranteed or endorsed by the publisher.

## Supplementary Material

Supplementary Figure 1 In-frame deletion of LuxS.

Supplementary Figure 2 The complementation of LuxS in the deletion mutant.

Supplementary Figure 3 Phylogenetic tree of the LuxS genes.

## References

[B1] Armando J. W. Boghigian B. A. Pfeifer B. A. (2012). LC-MS/MS quantification of short-chain acyl-CoA’s in *Escherichia coli* demonstrates versatile propionyl-CoA synthetase substrate specificity. *Lett. Appl. Microbiol.* 54 140–148. 10.1111/j.1472-765X.2011.03184.x 22118660

[B2] Bassler B. L. Wright M. Showalter R. E. Silverman M. R. (1993). Intercellular signalling in *Vibrio harveyi*: sequence and function of genes regulating expression of luminescence. *Mol. Microbiol.* 9 773–786. 10.1111/J.1365-2958.1993.TB01737.X 8231809

[B3] Besemer J. Lomsadze A. Borodovsky M. (2001). GeneMarkS: a self-training method for prediction of gene starts in microbial genomes. Implications for finding sequence motifs in regulatory regions. *Nucleic Acids Res.* 29 2607–2618. 10.1093/nar/29.12.2607 11410670 PMC55746

[B4] Bodor A. Elxnat B. Thiel V. Schulz S. Wagner-Döbler I. (2008). Potential for luxS related signalling in marine bacteria and production of autoinducer-2 in the genus *Shewanella*. *BMC Microbiol.* 8:13. 10.1186/1471-2180-8-13 18215278 PMC2233627

[B5] Chaumeil P. A. Mussig A. J. Hugenholtz P. Parks D. H. (2020). GTDB-Tk: a toolkit to classify genomes with the genome taxonomy database. *Bioinformatics* 36 1925–1927. 10.1093/bioinformatics/btz848 31730192 PMC7703759

[B6] Chi W. Zheng L. He C. Han B. Zheng M. Gao W. (2017). Quorum sensing of microalgae associated marine *Ponticoccus* sp. PD-2 and its algicidal function regulation. *AMB Express* 7:59. 10.1186/S13568-017-0357-6 28281272 PMC5344870

[B7] Chin C. S. Peluso P. Sedlazeck F. J. Nattestad M. Concepcion G. T. Clum A. (2016). Phased diploid genome assembly with single-molecule real-time sequencing. *Nat. Methods* 13 1050–1054. 10.1038/nmeth.4035 27749838 PMC5503144

[B8] Cui X. Harling R. (2005). N-acyl-homoserine lactone-mediated quorum sensing blockage, a novel strategy for attenuating pathogenicity of Gram-negative bacterial plant pathogens. *Eur. J. Plant Pathol.* 111 327–339. 10.1007/S10658-004-4891-0

[B9] Ding N. Du W. Feng Y. Song Y. Wang C. Li C. (2021). Algicidal activity of a novel indigenous bacterial strain of *Paracoccus homiensis* against the harmful algal bloom species, *Karenia mikimotoi*. *Arch. Microbiol.* 203 4821–4828. 10.1007/S00203-021-02468-3 34212209

[B10] Dow L. (2021). How do quorum-sensing signals mediate algae–bacteria interactions? *Microorganisms* 9:1391. 10.3390/MICROORGANISMS9071391 34199114 PMC8307130

[B11] Gao W. Liu Y. Giometti C. S. Tollaksen S. L. Khare T. Wu L. (2006). Knock-out of SO1377 gene, which encodes the member of a conserved hypothetical bacterial protein family COG2268, results in alteration of iron metabolism, increased spontaneous mutation and hydrogen peroxide sensitivity in *Shewanella oneidensis* MR-1. *BMC Genomics* 7:76. 10.1186/1471-2164-7-76 16600046 PMC1468410

[B12] Guo X. Liu X. Wu L. Pan J. Yang H. (2016). The algicidal activity of *Aeromonas* sp. Strain GLY-2107 against bloom-forming *Microcystis aeruginosa* is regulated by N-acyl homoserine lactone-mediated quorum sensing. *Environ. Microbiol.* 18 3867–3883. 10.1111/1462-2920.13346 27105123

[B13] Huisman J. Codd G. A. Paerl H. W. Ibelings B. W. Verspagen J. M. H. Visser P. M. (2018). Cyanobacterial blooms. *Nat. Rev. Microbiol.* 16 471–483. 10.1038/s41579-018-0040-1 29946124

[B14] Kovach M. E. Elzer P. H. Steven Hill D. Robertson G. T. Farris M. A. Roop R. M. (1995). Four new derivatives of the broad-host-range cloning vector pBBR1MCS, carrying different antibiotic-resistance cassettes. *Gene* 166 175–176. 10.1016/0378-1119(95)00584-1 8529885

[B15] Leangapichart T. Hadjadj L. Gautret P. Rolain J. M. (2021). Comparative genomics of two *Shewanella xiamenensis* strains isolated from a pilgrim before and during travels to the Hajj. *Gut Pathog.* 13:9. 10.1186/S13099-021-00404-W 33563327 PMC7871542

[B16] Li Z. Lin S. Liu X. Tan J. Pan J. Yang H. (2014). A freshwater bacterial strain, *Shewanella* sp. Lzh-2, isolated from Lake Taihu and its two algicidal active substances, hexahydropyrrolo[1,2-a]pyrazine- 1,4-dione and 2, 3-indolinedione. *Appl. Microbiol. Biotechnol.* 98 4737–4748. 10.1007/s00253-014-5602-1 24566920

[B17] Liébana R. Arregui L. Santos A. Murciano A. Marquina D. Serrano S. (2016). Unravelling the interactions among microbial populations found in activated sludge during biofilm formation. *FEMS Microbiol. Ecol.* 92:fiw134. 10.1093/femsec/fiw134 27306553

[B18] Lowe T. M. Eddy S. R. (1997). tRNAscan-SE: a program for improved detection of transfer RNA genes in genomic sequence. *Nucleic Acids Res.* 25 955–964. 10.1093/nar/25.5.955 9023104 PMC146525

[B19] Lu L. Niu X. Zhang D. Ma J. Zheng X. Xiao H. (2021). The algicidal efficacy and the mechanism of *Enterobacter* sp. EA-1 on oscillatoria dominating in aquaculture system. *Environ. Res.* 197:111105. 10.1016/J.ENVRES.2021.111105 33839120

[B20] Mukherjee M. Zaiden N. Teng A. Hu Y. Cao B. (2020). Shewanella biofilm development and engineering for environmental and bioenergy applications. *Curr. Opin. Chem. Biol.* 59 84–92. 10.1016/j.cbpa.2020.05.004 32750675

[B21] Nguyen N. T. Takemura T. Pham A. H. Q. Tran H. T. Vu K. C. T. Tu N. D. (2020). Whole-genome sequencing and comparative genomic analysis of *Shewanella xiamenensis* strains carrying blaOXA-48-like genes isolated from a water environment in Vietnam. *J. Glob. Antimicrob. Resist.* 21 272–274. 10.1016/J.JGAR.2020.04.033 32387641

[B22] Pal M. Purohit H. J. Qureshi A. (2021). Genomic insight for algicidal activity in Rhizobium strain AQ_MP. *Arch. Microbiol.* 203 5193–5203. 10.1007/s00203-021-02496-z 34341843

[B23] Saltikov C. W. Newman D. K. (2003). Genetic identification of a respiratory arsenate reductase. *Proc. Natl. Acad. Sci. U.S.A.* 100 10983–10988. 10.1073/pnas.1834303100 12939408 PMC196913

[B24] Sugita H. Kitao S. Narisawa S. Minamishima R. Itoi S. (2017). Diversity of culturable bacterial communities in the intestinal tracts of goldfish (*Carassius auratus*) and their ability to produce N-acyl homoserine lactone. *Folia Microbiol. (Praha).* 62 263–267. 10.1007/s12223-017-0498-7 28124783

[B25] Tamura K. Stecher G. Kumar S. (2021). MEGA11: molecular evolutionary genetics analysis version 11. *Mol. Biol. Evol.* 38 3022–3027. 10.1093/MOLBEV/MSAB120 33892491 PMC8233496

[B26] Tian C. Liu X. Tan J. Lin S. Li D. Yang H. (2012). Isolation, identification and characterization of an algicidal bacterium from Lake Taihu and preliminary studies on its algicidal compounds. *J. Environ. Sci.* 24 1823–1831. 10.1016/S1001-0742(11)60983-2 23520853

[B27] Walker B. J. Abeel T. Shea T. Priest M. Abouelliel A. Sakthikumar S. (2014). Pilon: an integrated tool for comprehensive microbial variant detection and genome assembly improvement. *PLoS One* 9:112963. 10.1371/JOURNAL.PONE.0112963 25409509 PMC4237348

[B28] Wu L. Guo X. Liu X. Yang H. (2017). NprR-NprX quorum-sensing system regulates the algicidal activity of *Bacillus* sp. Strain S51107 against bloom-forming cyanobacterium *Microcystis aeruginosa*. *Front. Microbiol.* 8:1968. 10.3389/FMICB.2017.01968 29075240 PMC5641580

[B29] Xue G. Wang X. Xu C. Song B. Chen H. (2021). Removal of harmful algae by *Shigella* sp. H3 and *Alcaligenes* sp. H5: algicidal pathways and characteristics. *Environ. Technol.* 10.1080/09593330.2021.1949047 34184617

[B30] Yang C. Hou X. Wu D. Chang W. Zhang X. Dai X. (2020). The characteristics and algicidal mechanisms of cyanobactericidal bacteria, a review. *World J. Microbiol. Biotechnol.* 36:188. 10.1007/s11274-020-02965-5 33241509

[B31] Ye W. Tan J. Liu X. Lin S. Pan J. Li D. (2011). Temporal variability of cyanobacterial populations in the water and sediment samples of Lake Taihu as determined by DGGE and real-time PCR. *Harmful Algae* 10 472–479. 10.1016/J.HAL.2011.03.002

[B32] Yousfi K. Touati A. Lefebvre B. Fournier É Côté J. C. Soualhine H. (2017). A novel plasmid, pSx1, harboring a new Tn1696 derivative from extensively drug-resistant *Shewanella xiamenensis* encoding OXA-416. *Microb. Drug Resist.* 23 429–436. 10.1089/mdr.2016.0025 27505638

[B33] Zhang C. Li Y. Meng C. X. Yang M. J. Wang Y. G. Cai Z. H. (2020). Complete genome sequence of Acinetobacter baumanni J1, a quorum sensing-producing algicidal bacterium, isolated from Eastern Pacific Ocean. *Mar. Genomics* 52:100719. 10.1016/J.MARGEN.2019.100719 31680055

[B34] Zhang Q. Wang Y. Zhou J. (2021). Complete genome sequence of *Stenotrophomonas* rhizophila KC1, a quorum sensing-producing algicidal bacterium, isolated from mangrove *Kandelia candel*. *Mol. Plant Microbe Interact.* 34 857–861. 10.1094/mpmi-12-20-0346-a 33673750

[B35] Zhang S. J. Du X. P. Zhu J. M. Meng C. X. Zhou J. Zuo P. (2020). The complete genome sequence of the algicidal bacterium *Bacillus subtilis* strain JA and the use of quorum sensing to evaluate its antialgal ability. *Biotechnol. Rep.* 25:e00421. 10.1016/J.BTRE.2020.E00421 31956522 PMC6961068

[B36] Zhao J. Quan C. Jin L. Chen M. (2018). Production, detection and application perspectives of quorum sensing autoinducer-2 in bacteria. *J. Biotechnol.* 268 53–60. 10.1016/J.JBIOTEC.2018.01.009 29355813

[B37] Zhu J. Huang X. Zhang F. Feng L. Li J. (2015). Inhibition of quorum sensing, biofilm, and spoilage potential in *Shewanella baltica* by green tea polyphenols. *J. Microbiol.* 53 829–836. 10.1007/s12275-015-5123-3 26626353

[B38] Zhu J. Zhao A. Feng L. Gao H. (2016). Quorum sensing signals affect spoilage of refrigerated large yellow croaker (*Pseudosciaena crocea*) by *Shewanella baltica*. *Int. J. Food Microbiol.* 217 146–155. 26519730 10.1016/j.ijfoodmicro.2015.10.020

