# Supplementary figures and images for: The LuxS/AI-2 Quorum-Sensing System Regulates the Algicidal Activity of Shewanella xiamenensis Lzh-2
Source: Front Microbiol. 2022 Jan 28;12:814929. doi: 10.3389/fmicb.2021.814929 (PMC8831721; doi:10.3389/fmicb.2021.814929)

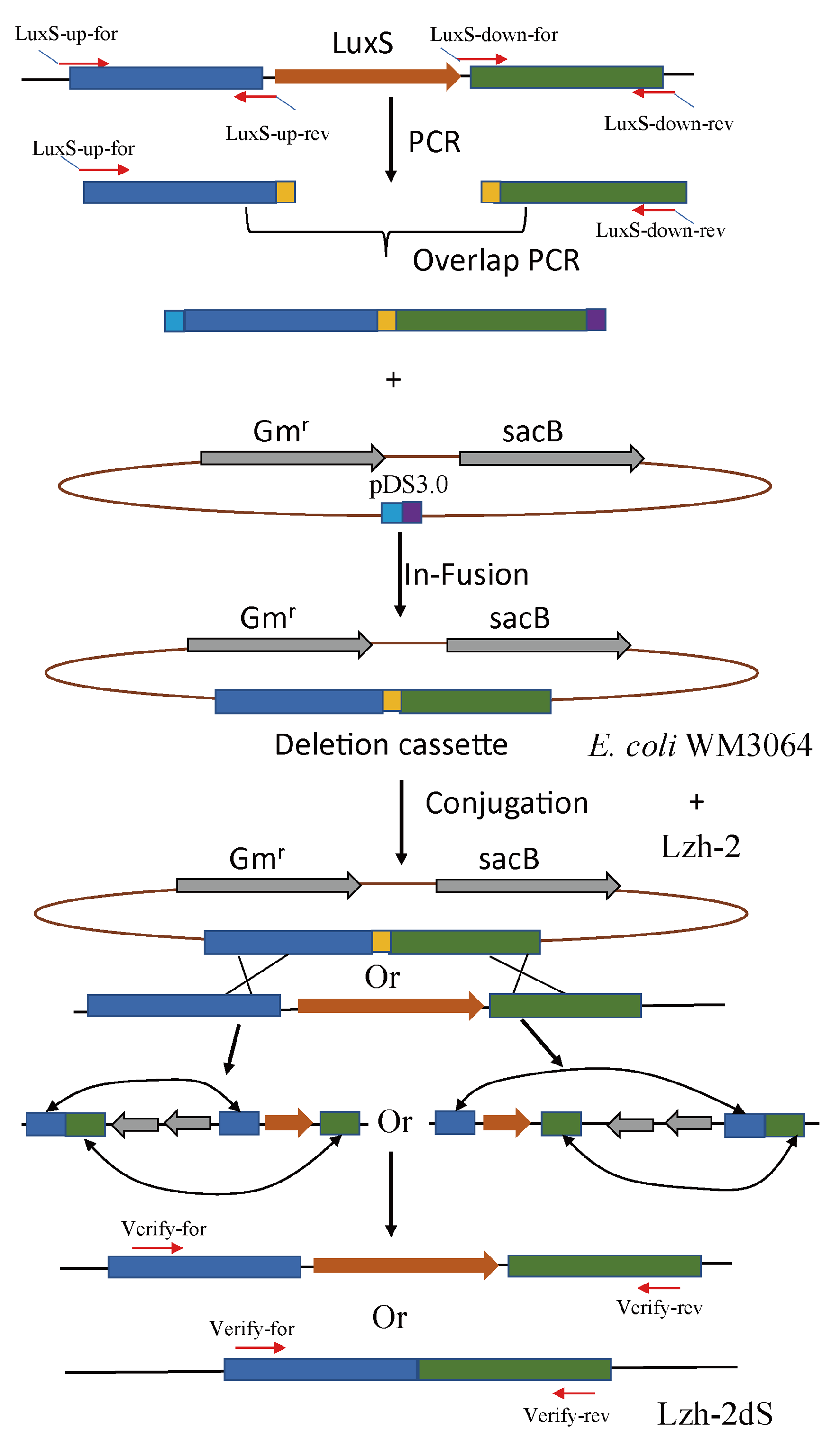

Supplement: Supplementary Figure 1 — In-frame deletion of LuxS. [file Image_1.TIF]

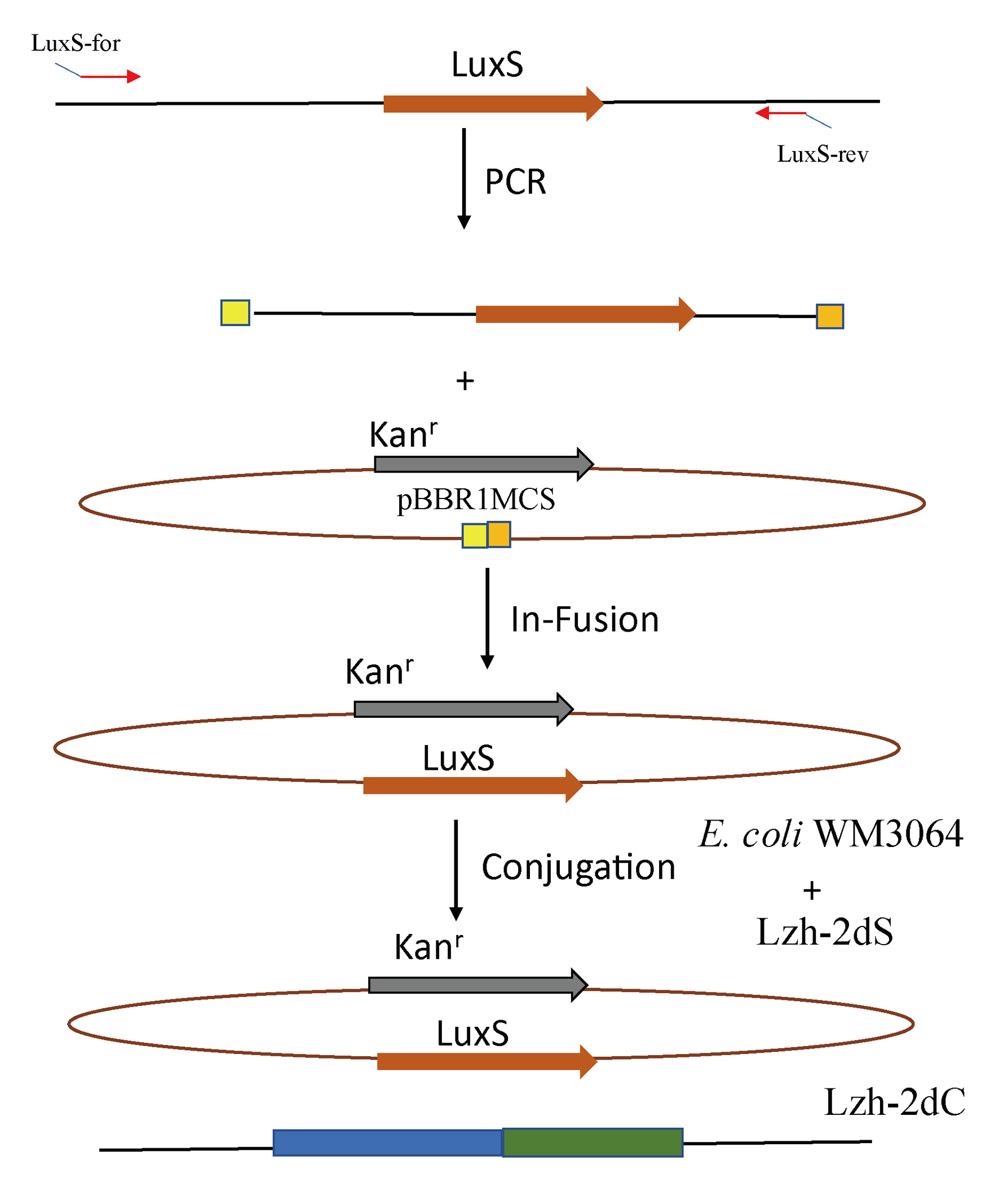

Supplement: Supplementary Figure 2 — The complementation of LuxS in the deletion mutant. [file Image_2.TIF]

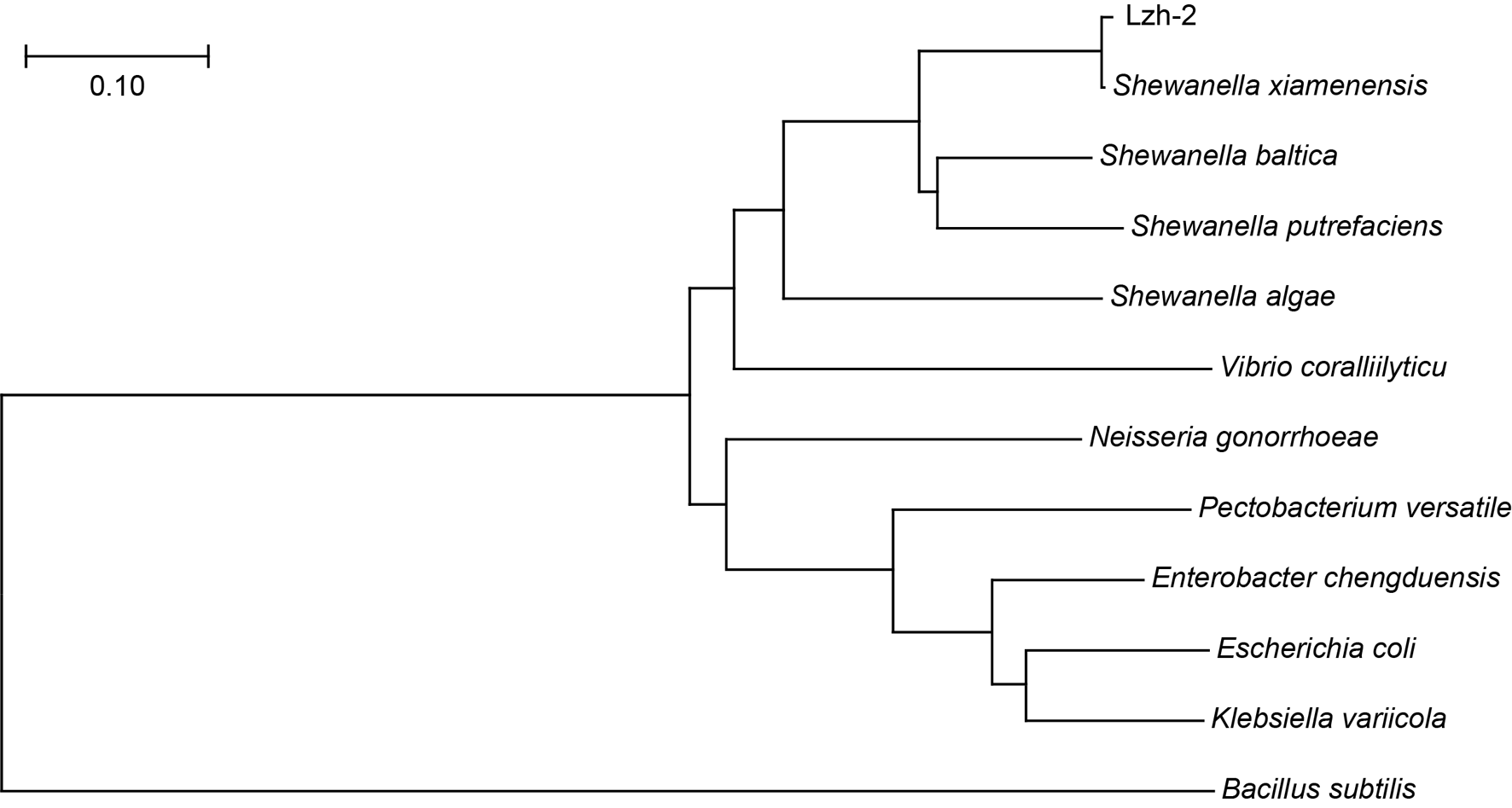

Supplement: Supplementary Figure 3 — Phylogenetic tree of the LuxS genes. [file Image_3.TIF]
