# Supplementary material for: The LuxS/AI-2 Quorum-Sensing System Regulates the Algicidal Activity of Shewanella xiamenensis Lzh-2
Source: Front Microbiol. 2022 Jan 28;12:814929. doi: 10.3389/fmicb.2021.814929 (PMC8831721; doi:10.3389/fmicb.2021.814929)
Supplement: Supplementary file 4 [file Table_1.DOC]

**Table S1** Strains and plasmids used in this study.

| **Strain or plasmid** | **Description** | **Reference or source** |
| --- | --- | --- |
| ***Escherichia coli* strain** |  |  |
| WM3064 | Donor strain for conjugation; ΔdapA | (Saltikov and Newman, 2003) |
| **Cyanobacterial** |  |  |
| *Microcystis aeruginosa* 9110 | Algicidal activity test | (Li et al., 2014) |
| ***Shewanella xiamenensis* strains** |  |  |
| LZH-2 | Wild-type | Li et al. 2014 |
| LZH-2dS | LuxS deletion mutant | This study |
| LZH-2dC | LuxS complementation | This study |
| **AI-2 bioassys** |  |  |
| *Vibrio harveyi* BB152 ATCC® BAA1119™ | Biosensor of AI-2 | (Bassler et al., 1993) |
| *V. harveyi* BB170 ATCC® BAA-1117™ | Positive control of AI-2 | (Bassler et al., 1993) |
| **Plasmids** |  |  |
| pDS3.0 | Apr, Gmr, derivative from suicide vector pCVD442 | Gao et al. 2006 |
| pDel-S | pDS3.0+LuxS deletion cassete | This study |
| pBBR1MCS-2 | Broad host Kanr vector used for complementation | Kovach et al. 1995 |
| pComp-S | pBBR1MCS-2+LuxS | This study |
