# Supplementary material for: The LuxS/AI-2 Quorum-Sensing System Regulates the Algicidal Activity of Shewanella xiamenensis Lzh-2
Source: Front Microbiol. 2022 Jan 28;12:814929. doi: 10.3389/fmicb.2021.814929 (PMC8831721; doi:10.3389/fmicb.2021.814929)
Supplement: Supplementary file 5 [file Table_2.DOC]

**Table S2** Primers used in this study.

| **Name** | **Sequence** |
| --- | --- |
| LuxS-up-for | tggaattcccgggagCGATGGCGTTAGCCAAGGT |
| LuxS-up-rev | gttaagcccatatttgcggcAGGGATCTCCAAATTATCAGGG |
| LuxS-down-for | ccctgataatttggagatccctgccgcaaatatgggcttaac |
| LuxSI-down-rev | ccgcatgcgatatcgCGCTAATCACTCGACTGTCAC |
| LuxS-for | tatagggcgaattggCCAGATGGCGCATGCAGCC |
| LuxS-rev | ccgccaccgcggtggGTAATGGCATAGGGATCTCC |
| Verify-for | GATGGCGTTAGCCAAGGT |
| Verify-rev | GCTAATCACTCGACTGTCA |
