# Supplementary material for: The LuxS/AI-2 Quorum-Sensing System Regulates the Algicidal Activity of Shewanella xiamenensis Lzh-2
Source: Front Microbiol. 2022 Jan 28;12:814929. doi: 10.3389/fmicb.2021.814929 (PMC8831721; doi:10.3389/fmicb.2021.814929)
Supplement: Supplementary file 6 [file Table_3.DOC]

**Table S3** Gene IDs of the selected LuxS genes in Genebank.

| **Organism** | Gene ID |
| --- | --- |
| *Bacillus subtilis* | 937106 |
| *Enterobacter chengduensis* | 63143757 |
| *Escherichia coli* | 947168 |
| *Klebsiella variicola* | 56937264 |
| *Neisseria gonorrhoeae* | 57106558 |
| *Pectobacterium versatile* | 57313255 |
| *Shewanella algae* | 61692371 |
| *Shewanella baltica* | 11773601 |
| *Shewanella putrefacien* | 45041370 |
| *Shewanella xiamenensis* | 58508280 |
| *Vibrio coralliilyticus* | 58276749 |
